# Preliminary Volumetric Calculation of the Mucosal Surface in the Nares of Lambs Using a Segmentation of Computed Tomographic Images

**DOI:** 10.3389/fvets.2020.620647

**Published:** 2020-12-18

**Authors:** K. P. Teeling, D. Werling, D. Berner

**Affiliations:** ^1^Department of Clinical Science and Services, Royal Veterinary College, Hatfield, United Kingdom; ^2^Department of Pathobiology and Population Sciences, Royal Veterinary College, Hatfield, United Kingdom

**Keywords:** mucosal surface, nasal volume, vaccination, aerosol, ruminant

## Abstract

Intranasal vaccinations are becoming more important in both human and animal medicine to generate a localized IgA immune response not seen with parenteral vaccinations. This localized IgA response is more effective at reducing pathogen load on the mucosal surface of a potential host. One prerequisite for a successful nasal vaccination is the need to understand the distribution pattern of the nebulized vaccine, which requires an understanding the volume of the nares as well as the mucosal surface area. The exact mucosal surface area of ruminant nares has not yet been investigated. The aim of this concept study is to provide a detailed breakdown of a new method of volumetric rendering that can be used to calculate the volume and mucosal surface area of ruminant nares from computed tomographic images. The program Seg 3D was used to perform semi-automatic segmentation of a CT scan of a 9-month-old lamb head. Threshold segmentation and manual segmentation were used in combination to select the lamb's nasal cavity. The segmentation process yielded a volumetric rendering that was used to calculate the surface area and volume of the lamb's nasal cavity, with the segmentation process was repeated for each individual side of the lamb's nares. The surface area of the mucosal surface of each nostril is approximately 448 cm^2^, and the volume is approximately 45 cm^3^. The methodology described in this study successfully calculated the volume and surface area of a lamb's nares using volumetric rendering.

## Introduction

Over the last decade, intranasal vaccines have been utilized more widely in human and animal medicine. Many drugs can be readily absorbed through the nasal mucosa, and the intranasal vaccinations currently in use have been found to be both more effective and safer than systemic vaccination (1, 2). The first human intranasal vaccine, FluMist™, was approved for use in the US in 2003, and there are records of intranasal vaccines being produced for cattle as early as 1974 (3). The most recent intranasal vaccine produced for cattle, Bovilis® Nasalgen® 3, was approved in the US this year (2020). These vaccines, independent of their use in humans or animals, were all approved based on efficacy and safety studies (4). Although this ensures the vaccines will produce, in general, a set minimum level of protection against the intended diseases, it can be difficult to accurately test immunity in a controlled research environment (5). When conducting these experiments, a concentrated dose of the pathogen is administered to vaccinated and control animals to measure their response to the challenge infection, usually *via* reduction of pathogen shedding and severity of clinical signs (5). However, under farm conditions animals are initially protected through maternal antibodies or in case of existing and consequently conducted vaccination schemes through existing herd-immunity. Under such circumstances, animals are only gradually exposed to the pathogen load outside of controlled research environments (5). Hence, in such circumstances, a vaccine may not need the same high dose to protect the animal against a natural infection as was needed to protect the animal in the controlled test environment used for vaccine approval before it is released onto the market (5). Despite this, the applied mucosal vaccine should still be taken up by as many immune cells underlying the mucosal surfaces as possible.

Intranasal vaccines were designed based on the concept of a common mucosal immune system. The common mucosal immune system is a concept that states IgA immune responses of the mucosal system can spread to other mucosal sites in the body through the migration of T and B cells *via* the lymphatic and circulatory systems (6). Studies have shown strong evidence for the existence of a common mucosal immune system (7). The immune response at other mucosal sites will be weaker initially compared to the response at the initial site of antigen or vaccine exposure, and there is also a time delay between antigen exposure in one location and immune response in another (6, 7). For this reason, it is very important that vaccines are given at the location where the strongest immune response will be required (6). Parenteral vaccines have not been found to trigger IgA production, whereas intranasal vaccines do trigger IgA production, and are currently administered as a single dose to one nostril (6–8). The aim of intranasal vaccinations is to reduce pathogen load on entry ports by stimulating the local mucosal-associated lymphatic tissue as well as prime for a subsequent systemic IgG response through the uptake of the vaccine antigen by M cells and mucosal dendritic cells (9), with both cell types constantly surveying the mucosal surfaces. In an efficacy study for Bovilis Nasalgen® 3 approval, 59% of the vaccinated calves exposed to Parainfluenza 3 shed the virus (10). Another study conducted on a Parainfluenza 3 vaccine in 1985, also found that vaccinated calves still shed the virus in their nasal secretions (8). Although this was considered significantly lower compared to the unvaccinated calves, a new approach involving vaccination of both nostrils may be more effective (6, 11).

One of the key elements of an effective intranasal vaccine is bioavailability (2). If the vaccine cannot be absorbed and utilized by the body, it will have no effect. Nasal volume and the device used to deposit the vaccine, in the form of a mist, have a large effect on the bioavailability of the product (1, 12). A study conducted by Gizurarson in 2012 found intranasal drug absorption occurs primarily *via* columnar cells in the nasal conchae and meatus, as a result mucosal surface area greatly affects intranasal vaccine absorption (13). Previous studies have measured the surface area of the nasal mucosa in humans to further improve the production of intranasal drugs and vaccines, but when applied to lamb nares this process has been limited to estimates based on microscopic measurements (13–15). No previous studies have been performed to precisely measure the mucosal surface areas in calves or lambs, therefore the aim is to provide a detailed breakdown of a new method of volumetric rendering that can be used to calculate the volume and mucosal surface area of ruminant nares from CT images. It was hypothesized volumetric rendering can be used to calculate the mucosal surface area of lamb nares from CT images.

## Materials and Methods

### Animal

Due to the lockdown in the UK based on governmental COVID-19 regulations, only one lamb head was obtained during the study period by the Royal Veterinary College post-mortem room from a lamb that was humanly euthanized. The lamb was healthy prior to slaughter, and the head was used in this study prior to use by the pathology lab. As the sample was ethically obtained for other purposes, ethical approval was not required for this study. The lamb was estimated to be 9 months old based on its teething status. The second molars had almost completely erupted, while the first incisor had not yet erupted. The head was 15 cm from nares to ethmoid, 7.12 cm from hard palate to nasal bone, and 11.25 cm maximum width at the zygomatic bone. The head was harvested at the caudal aspect of the calvarium. The lamb head was stored frozen and defrosted 1 day before the scan.

### Computed Tomography Scan

A LightSpeed RT 16 (GE Medical Systems, UK) was used to obtain a CT scan of the lamb head on April 28, 2020. Images were acquired using 120 kV, 255 mAs, slice thickness of 1.25 mm, helical mode, and a pitch of 0.9375. The head was placed in ventral recumbency. A bone reconstruction was performed using a high frequency filter, a slice thickness of 0.65 mm with an overlap of 0.3 mm, and a FOV was 193 mm. The images were compared to a study on surgical anatomy of *Ovis aries* for normal anatomy and pathology (16). Fluid was found in the right nasal cavity on transverse slide 496 of 701.

### Calculation of Mucosal Surface Area and Volume

The methodology applied in the present study was based on previous publications (12, 17, 18) and modified as follows. The form of semi-automated segmentation used in these studies was connected neighborhood segmentation, also known as region growing, which begins in a seeded location and identifies nearby pixels that fit the same criteria as the initial input (18, 19). The program will grow the selected region from the seeded location until there are no nearby pixels that fit the criteria of the initial input (19). Connected neighborhood segmentation was attempted, however the 2016 Lenovo ThinkPad E560 used for this study was unable to complete the program, as the segmentation methods available for use *via* the CIBC Seg3D2 Segmentation software (Seg 3D, version 2.4.4) are limited to the amount of RAM the computer has available.

The CT image series was imported into the Seg 3D software developed by the University of Utah Scientific Computing and Imaging Institute (SCI). Semi-automated segmentation was used to select the nasal cavity of the lamb head. Threshold segmentation was used as a substitute for connected neighborhood segmentation. Threshold segmentation relies on homogeneity and will select pixels that fall within a chosen range (19).

The nasal cavity was sampled for the range of “values” present to create a desired range for the threshold tool. The first range attempted was −3024 to −890.75. After this, the range −1073.6 to −768.85 was used before settling on the final range of −1073.6 to −525.05. The decision was made to over-select the desired area and refine the selection with manual segmentation rather than to miss vital data. As the threshold tool selects all areas that fall within a range of values, the sinus cavities and some areas outside of the head were selected. The incisive bones and the hard palate were used as landmarks for where the nasal mucosa began and ended, respectively. This correlated to transverse slides 392 to 1.

The crop tool was used to remove as much of the excess selection as possible to speed up the process of manual selection. This was done three separate times until the selected area was close to the desired data range without removing any of the data. The detailed manual selection was completed using the polyline tool. The areas where the threshold mask selected undesired data were removed from the selection in careful detail. The manual segmentation was regularly checked by a second researcher to ensure accuracy. This initial selection process took 336 h.

A volumetric rendering was created for a 3D visualization of the lamb's nasal cavity. Previous studies utilized unconstrained smoothing to refine the surface of their volumetric rendering (12, 17, 18); however, Raffan et al. (20) found unconstrained smoothing removed finite details in 3D renderings of small structures (20). Since the aim of the present study was to focus on surface detail and area, the decision was made not to use unconstrained smoothing for this study. The volume and surface area of the volumetric rendering were calculated using the Seg3D software. The surface area of the volumetric rendering is the mucosal surface area of the nasal cavity, expressed as the measurements found in the original imported files, which in the present study was millimeters.

Since intranasal vaccinations often are given into a single nostril, the volume and surface area of each nostril was calculated. The crop tool was used twice to create two new layers that each primarily covered one half of the lamb's nares. Due to the angle of the initial CT scan, manual segmentation was required to ensure the two new layers each covered only one half of the lamb's nares. The right nostril was segmented first. The polyline tool was used to remove any additional selection until the layer entitled “Right Side” only selected the right nostril. The polyline tool was then used to manually segment the layer entitled “Left Side.” In cases where there was not a physical separation between the two sides visible on the CT scan, the midline was used as an anatomical marker. The field of view was zoomed in when segmenting these areas to ensure the two sides met in a straight line without overlapping. This manual segmentation took 35 h. Volumetric renderings were created for both of the nares, and the surface area and volume of each were recorded.

## Results

### Volumetric Rendering

**Figure 1:**
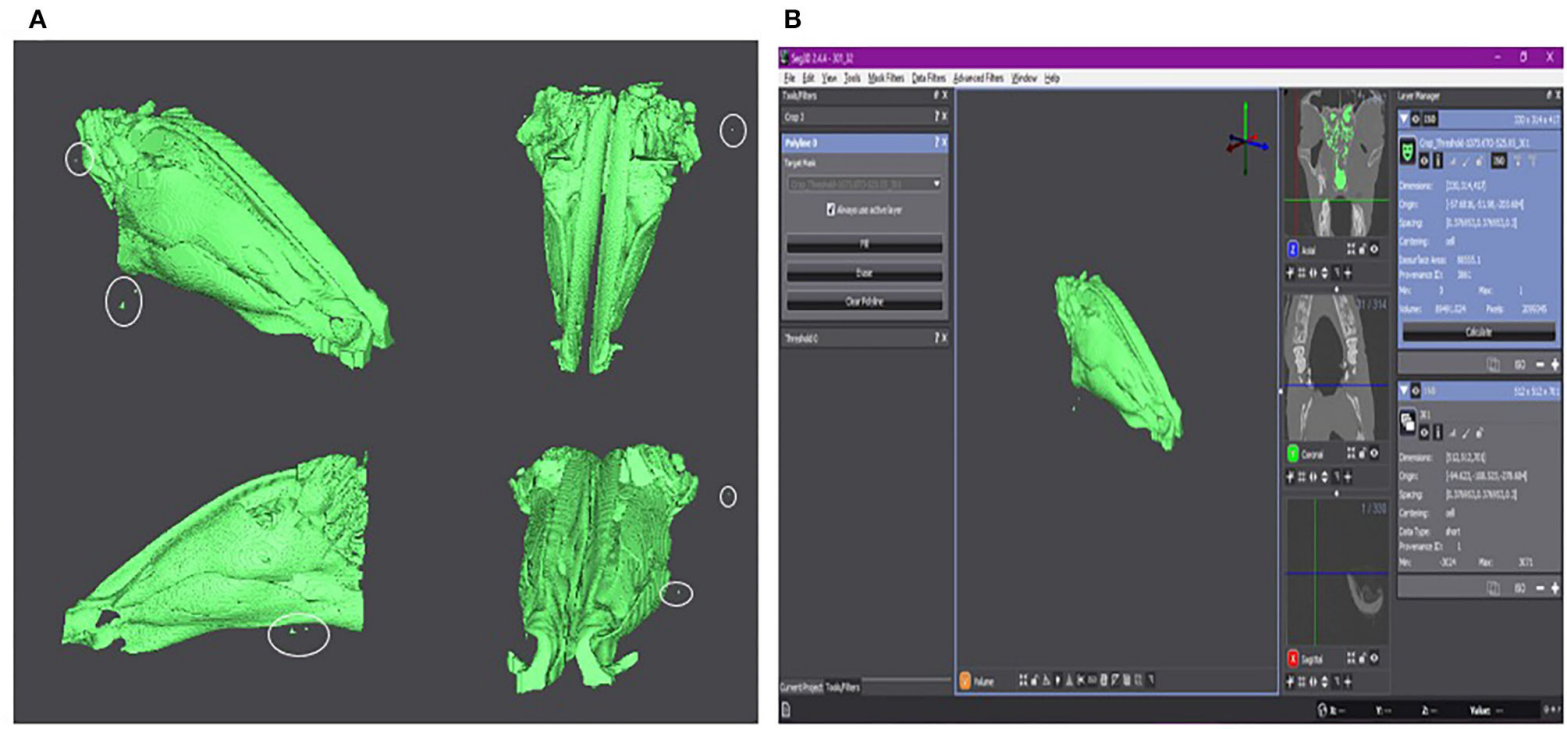
**(A)** The volumetric rendering of the lamb's nares. Isoselesis (top left). Dorsoventral view (top right). Lateral view (bottom left). Rostrocaudal view (bottom right). Outliers are marked by a white circle. **(B)** Screen shot of the Seg 3D program with the isoselesis view of volumetric rendering in the center panel. The three tools used for the semi-automatic segmentation of the CT scan can be seen in the left panel. To the right of the central panel is the three imaging planes. The right-most panel shows the two final layers at this stage of the study. The layer “Crop_Threshold-1073.6TO-525.05_301” is highlighted and shows the surface area and volume of the volumetric rendering. The layer “301” is the initial CT scan that was used to create the segmentation layer visible in light green.

The volumetric rendering of the nares can be seen in Figure 1A, with Figure 1B showing a screen shot of the Seg 3D program with the calculated surface area and volume. As seen in both figures, there are outliers selected in the volumetric rendering (marked by white circles in Figure 1A). These areas could not be removed from the selection but should not significantly affect the final results.

**Figure 2:**
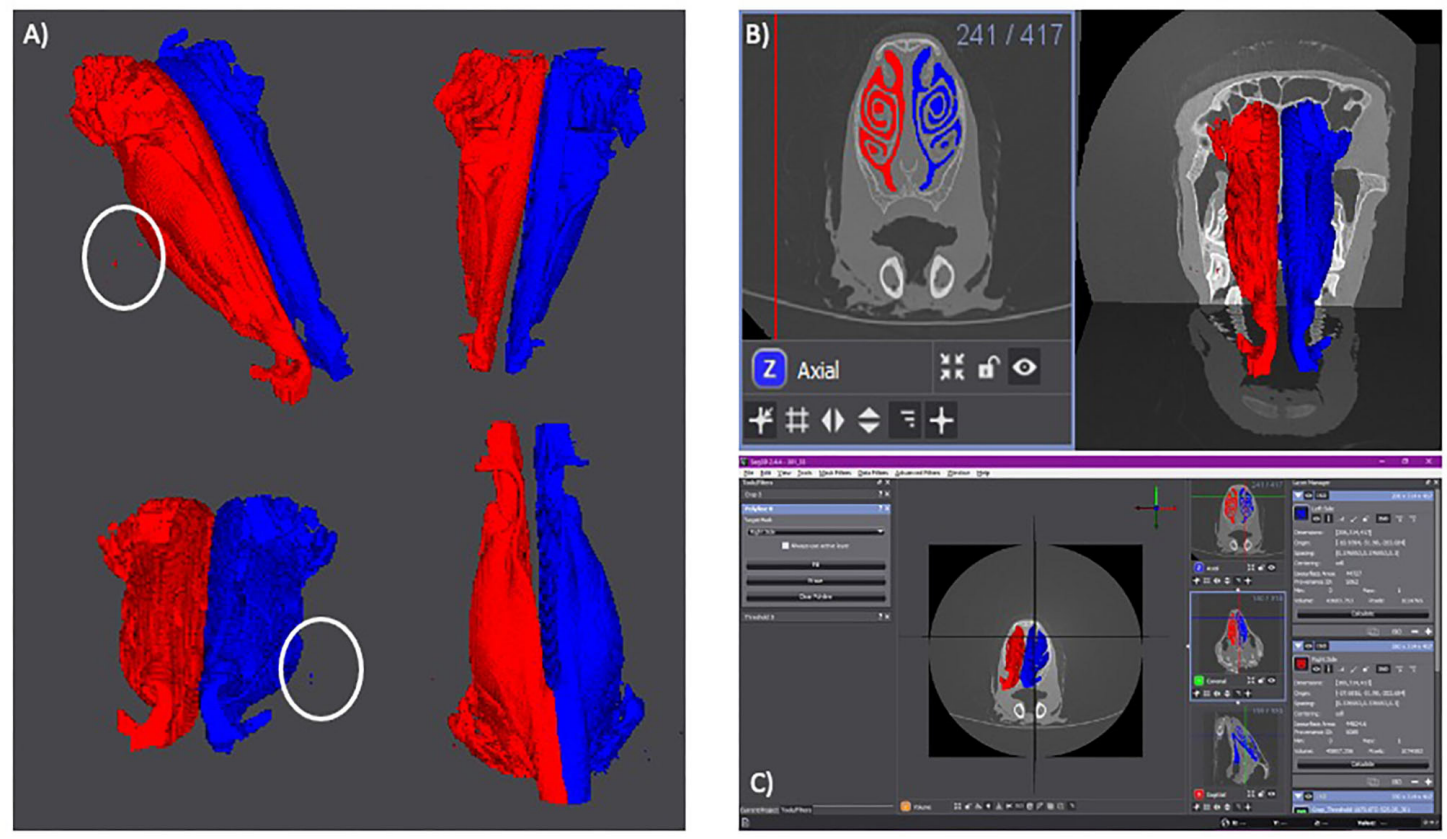
**(A)** Volumetric rendering of each side of the lamb's nares. The right side is in red. The left side is in blue. Isoselesis view (top left). Dorsoventral view (top right). Rostrocaudal view (bottom left). Ventrodorsal view (bottom right). Outliers are marked by a white circle. **(B)** Axial slide 241 with the right (red) and left (blue) sides of the lamb's nares selected (left). Volumetric rendering of the right and left nares with the CT scan planes visible for anatomical reference (right). **(C)** Screen shot of the Seg 3D program with the anteroposterior view of the volumetric rendering in the center panel. The three tools used for the semi-automatic segmentation can be seen in the left panel. To the right of the central panel is the three imaging planes. The right-most panel shows the “Right Side” layer (red) and the “Left Side” layer (blue). These two layers show the surface area and volume of their individual volumetric renderings.

The results of the volumetric renderings of the right and left nares can be seen in Figure 2A, with the outliers visible in Figure 1 being present in both, the left and right nostrils. There are more outliers present on the right side than the left, however the outliers should not affect the final results.

The selected areas of each nostril and the volumetric rendering in relation to the original CT scans are shown in Figure 2B, with Figure 2C showing a screen shot of the Seg 3D program with the calculated surface area and volume for the right and left nostrils.

### Calculated Surface Area and Volume

The surface area of the mucosal surface and volume of the lamb's nasal cavity can be seen in Table 1. The sum of the surface areas for the right and left nostrils is 895.52 cm^2^, which is larger than the calculated value of the entire nasal cavity surface area by 9.965 cm^2^. This increase in value occurs due to the surface area calculations for each individual nostril including the midline where the two sides meet, which is excluded from the entire nasal cavity surface area. The sum of the volumes for the left and right nostrils is 89.5 cm^3^, which is equal to the calculated value of the entire nasal cavity.

**Table 1:** Mucosal surface area and volume of the lamb's nares calculated using volumetric rendering.

**Anatomical location**	**Surface area (cm^**2**^)**	**Volume (cm^**3**^)**
Right nostril	448.246	45.8074
Left nostril	447.27	43.6838
Entire nasal cavity	885.551	89.491

## Discussion

The aim of this study was to provide a detailed breakdown of a new method of volumetric rendering that can be used to calculate the volume and mucosal surface area of ruminant nares. No previous studies have been performed to precisely measure the mucosal surface areas in ruminants. Previous studies have shown each human nostril has a volume of approximately 11.5 cm^3^ (12) and equine sinus volume ranges from 589.84 to 2217.49 cm^3^ depending on breed and age (17). Due to the difference in anatomical structure between ruminants, humans, and equines, the nostril and sinus volumes are not comparable between species.

We are fully aware and cannot stress enough that the study is limited due to the use of only one head, which was the result of a complete lockdown due to COVID-19. However, we believe that the data generated are of interest from an anatomical point of view as well as providing vaccinologists with some data regarding the mucosal surface area and volume of ruminants nares with same size nares for nasal vaccine delivery. Furthermore, the results of this study provide a proof of concept for the effectiveness of the methods described. A volumetric rendering was created using a combination of threshold and manual segmentation. This volumetric rendering was used to successfully calculate the mucosal surface area and volume of the lamb's nares.

The above data was gathered using semi-automatic segmentation based on threshold segmentation. Threshold segmentation is the most basic form of automatic segmentation developed but was necessary due to the RAM available (19), and it became clear that future studies need to take enough computer power into account to perform the segmentation, allowing for even more detailed analysis, such as connected neighborhood or active contour segmentation to be performed. Connected neighborhood segmentation uses a seeded location to identify nearby pixels that fit the same criteria and grows the segmented area until the nearby pixels no longer fit the given criteria (19), whereas active contours use edge detection and manual input from a selected slice to segment nearby slices (19). It is believed active contours may be the ideal segmentation method for the nasal cavity as it allows for border identification, this will be ideal for the nasal cavity as it is open to the outside of the body. However, connected neighborhood segmentation is a proven method when segmenting nasal sinuses and should not be overlooked (12, 17, 18).

Due to the nature of threshold segmentation, this study relied on manual segmentation. However, there is no statistically significant difference between volumes that were calculated using manual and semi-automatic segmentation (21). Manual segmentation does require more hours of work than semi-automatic segmentation methods (22). In the future, when using a larger sample size, it will be imperative to employ a more time efficient method. Both training and practice working with the segmentation program improve the speed and accuracy of the results (22). In addition, Shi et al. (23) successfully used automatic segmentation without the need for manual correction by designing a program that defined the surface shape of the sinuses for automatic segmentation (23). This method should be considered for future studies with large sample sizes.

One component that should be included in future studies for confirmational purposes is the *in vitro* measurement of the nasal cavity volume to ensure accuracy of the methodology (19). One method of doing so would be injecting silicone directly into the nasal cavity after performing the CT scans (24). This allows the researcher to calculate a rough estimate of the volume of the nasal cavity easily and quickly. The value can then be used to verify the accuracy of the segmentation once all of the data has been gathered.

We believe that such data are important to obtain to allow for an efficacious delivery of intra-nasal vaccines. Given that there is a need to reduce the usage of antimicrobials in food producing animals, there is also a need to develop vaccines that can reduce pathogen load as well as prime for a good immune response (mucosal as well as systemic). Although specific mechanisms between nanoparticles and biological membranes are still under investigation, physical parameters such as particle size and shape, as well as biological tissue distribution, including mucociliary clearance, influence the protection and delivery of antigens to the site of action and uptake by target cells (25). Several factors need to be considered for improved mucosal vaccine development, including the delivery systems for the right-place and right-time vaccine delivery, and we believe that an understanding of the volume and surface area that should be covered will help new nebulizers to be developed and nanoparticle size adjusted to optimize their distribution.

## Conclusion

The goal of this study was to provide a detailed breakdown of a new method of volumetric rendering that can be used to calculate the volume and mucosal surface area of ruminant nares. The volumetric renderings created using the Seg 3D program successfully provided the volume and surface area of the lamb's nares. Due to the limited sample size, the method described will have to be further corroborated by a future study with a larger sample size. It is recommended that these studies utilize either active contour or connected neighborhood segmentation to improve the speed and accuracy of the segmentation process. The data gathered using the described method of volumetric rendering can be used to further improve the dosage and efficacy of intranasal vaccinations.

## Data Availability Statement

The raw data supporting the conclusions of this article will be made available by the authors, without undue reservation.

## Ethics Statement

Ethical review and approval was not required for the animal study because a head obtained from the post-mortem room was used for the present study.

## Author Contributions

DW and DB developed the idea and approach. KT and DB performed investigations. All authors contributed to the article and approved the submitted version.

## Conflict of Interest

The authors declare that the research was conducted in the absence of any commercial or financial relationships that could be construed as a potential conflict of interest.
